# Supplementary material for: Molecular dynamics study of differential effects of serotonin-2A-receptor (5-HT2AR) modulators
Source: PLoS Comput Biol. 2025 Sep 3;21(9):e1013000. doi: 10.1371/journal.pcbi.1013000 (PMC12443254; doi:10.1371/journal.pcbi.1013000)
Supplement: S1 Table — Definition of the collective variables (CVs) in order to preserve the geometry of the G protein construct (with atom colors corresponding those in S1 Fig). Each CV is restrained using a flat-bottom harmonic potential, where the third column specifies the range within which no force is applied, and the force constant applied outside this range is provided in the last column. (DOCX) [file pcbi.1013000.s001.docx]

**S1 Table.** Definition of the collective variables (CVs) in order to preserve the geometry of the G protein construct (with atom colors corresponding those in Fig. S1). Each CV is restrained using a flat-bottom harmonic potential, where the third column specifies the range within which no force is applied, and the force constant applied outside this range is provided in the last column.

| CV atom selection | Atom color in Fig. S1 | Distance (Å) | Force constant |
| --- | --- | --- | --- |
| C_α_ resid 20  C_α_ resid 34 | Green | 24.2 – 25.2 | 2 |
| C_α_ resid 22  C_α_ resid 29 | Pink | 10.4 – 11.4 | 2 |
| O resid 34  H resid 82 | Cyan | 0 – 2 | 0.5 |
| H resid 38  O resid 84 | Purple | 0 – 2 | 0.5 |
| H resid 85  O resid 70 | Ice blue | 0 – 2 | 0.5 |
