# Supplementary material for: Molecular dynamics study of differential effects of serotonin-2A-receptor (5-HT2AR) modulators
Source: PLoS Comput Biol. 2025 Sep 3;21(9):e1013000. doi: 10.1371/journal.pcbi.1013000 (PMC12443254; doi:10.1371/journal.pcbi.1013000)
Supplement: S2 Table — Attributes used for the PCA. When multiple atoms or a range of residues are selected, the coordinates of the geometric mean of the non-H-atom is used for that DOF. [c] A representative frame of an inactive conformation with zotepine (simulation 1) is used as reference. [d] The cryoEM with pdb entry 6WHA was used as reference. (DOCX) [file pcbi.1013000.s002.docx]

**S2 Table.** Attributes used for the PCA. When multiple atoms or a range of residues are selected, the coordinates of the geometric mean of the non-H-atom is used for that DOF. [a] A representative frame of an inactive conformation with zotepine (simulation 1) is used as reference. [b] The cryoEM with pdb entry 6WHA was used as reference.

| PCA attribute label | Internal degree of freedom | atom selection |
| --- | --- | --- |
| TM26 | Outward movement TM6 | Distance centroids of CA of resid 109-115 and 319-325 |
| TM37 | Inward movement TM3 and TM7 | Distance centroids of Cas of resid 167-173 and 377-383 |
| Ionic lock | Ionic lock breaks between R3x50 and E6x30 | Distance centroids of both protonatable groups of resid 173 and 318 |
| TM23 | Movement of TM3 relative to TM2 | Distance centroids of CA of resid 109-115 and 167-173 |
| TM27 | Movement of TM7 relative to TM2 | Distance centroids of CA of resid 109-115 and 377-383 |
| TM36 extra | Extracellular distance TM3 and TM6 | Distance centroids of Cas of resid 148-154 and 342-348 |
| TM36 intra | Intracellular distance TM3 and TM6 | Distance centroids of Cas of resid 148-154 and 319-325 |
| TM46 | Movement of TM6 relative to TM4 | Distance centroids of Cas of resid 191-197 and resid 319-325 |
| TM47 | Movement of TM7 relative to TM4 | Distance centroids of Cas of resid 191-197 and resid 377-383 |
| TM56 extra | Ligand dependent interaction Leu236^5x41^ and Ile348^6x60^ | Distance centroids of resid 236 and 348 |
| TM56 intra | Intracellular distance between TM5 and TM6 | Distance centroids of Cαs of resid 256-262 and 319-325 |
| TM67 | Intracellular distance between TM6 and TM7 | Distance centroids of Cαs of resid 319-325 and 377-383 |
| I3x46 L6x37 | Hydrophobic interaction Ile169 and Leu325 | Distance centroids of resid 169 and 325 |
| I3x40 F6x44 | distance Ile163 and Phe332 of the PIF motif | Distance centroids of resid 163 and 325 |
| F34x56 R3x50 | Distance Phe186 (ICL2) and Arg173 (ionic lock) | Distance centroids of resid 186 and 173 |
| P34x50 I3x40 | Distance Pro180 (ICL2) and Ile163 (PIF) | Distance centroids of resid 180 and 163 |
| TyrTyr | Distance Tyr254 and Tyr380 (NPxxY) | Distance centroids of resid 254 and 380 |
| TrpAsn | Distance Trp336 and Asn376 (NPxxY) | Distance between indole N of 336 and amide O of Asn side chain of 376 |
| S5x46 G7x41 | Distance S242 to G369 to measure TM5 bulge | Distance centroids of resid 242 and 369 |
| χ1 Y7x53 | χ1 dihedral angle of Tyr380 (NPxxY) | Dihedral angle of C – Cα – Cβ – Cγ of resid 380 |
| Χ2 Y7x53 | Χ2 dihedral angle of Tyr380 (NPxxY) | Dihedral angle of Cα – Cβ – Cγ – Cδ of resid 380 |
| χ1 W6x48 | χ1 dihedral angle of Trp336 (Trp switch) | Dihedral angle of C – Cα – Cβ – Cγ of resid 336 |
| χ2 W6x48 | χ2 dihedral angle of Trp336 (Trp switch) | Dihedral angle of Cα – Cβ – Cγ – Cδ of resid 336 |
| χ1 F6x44 | χ1 dihedral angle of Phe332 (PIF motif) | Dihedral angle of C – Cα – Cβ – Cγ of resid 332 |
| χ2 F6x44 | χ2 dihedral angle of Phe332 (PIF motif) | Dihedral angle of Cα – Cβ – Cγ – Cδ of resid 332 |
| RMSD inactive^[a]^ | RMSD relative to inactive conformation | Cα of resid 71-265 and 313-397 |
| RMSD PIF inactiv^[a]^ | RMSD PIF relative to inactive conformation | Resid 246, 163 and 332 |
| RMSD NPxxY inactiv^[a]^ | RMSD NPxxY relative to inactive conformation | Resid 376, 377 and 380 |
| RMSD active^[b]^ | RMSD relative to active conformation | Cα of resid 71-265 and 313-397 |
| RMSD NPxxY activ^[b]^ | RMSD NPxxY relative to active conformation | Resid 376, 377 and 380 |
| RMSD PIF active^[b]^ | RMSD PIF relative to inactive conformation | Resid 246, 163 and 332 |
| RMSD ICL2 activ^[b]^ | RMSD of ICL2 relative to active ICL2 helix | Resid 177 to 190 |
