# Supplementary material for: Molecular dynamics study of differential effects of serotonin-2A-receptor (5-HT2AR) modulators
Source: PLoS Comput Biol. 2025 Sep 3;21(9):e1013000. doi: 10.1371/journal.pcbi.1013000 (PMC12443254; doi:10.1371/journal.pcbi.1013000)
Supplement: S3 Table — These frames were excluded based on RMSD time series (S3-S4 Figs) to ensure that only equilibrated segments of the trajectories were used for visualization (e.g., PCA and density plots). (DOCX) [file pcbi.1013000.s003.docx]

**S3 Table. Overview of the initial portions of each simulation (in nanoseconds) that were discarded.** These frames were excluded based on RMSD time series (Figs S3-4) to ensure that only equilibrated segments of the trajectories were used for visualization (e.g., PCA and density plots).

| **Ligand** | **Non-G-protein coupled systems** | | | **G-protein coupled systems** | | |
| --- | --- | --- | --- | --- | --- | --- |
|  | Sim no | a | b (ns) | Sim no | a (ns) | b (ns) |
| **Zotepine** | 1 | 500 |  | 9 | 250 | 500 |
| **Apo** | 2 | 2200 | 200 | 10 | 500 | 500 |
| **Risperidone** | 3 | 500 |  |  |  |  |
| **Lisuride** | 4 | 250 | 250 | 11 | 200 | 200 |
| **(R)-69** | 5 | 600 | 1000 | 12 | 300 | 400 |
| **IHCH-7086** | 6 | 1000 |  | 13 | 500 | 250 |
| **25CN-NBOH** | 7 | 1000 |  | 14 | 250 | 250 |
| **LSD** | 8 | 250 | 200 | 15 | 200 | 200 |
