# Supplementary figures and images for: Molecular dynamics study of differential effects of serotonin-2A-receptor (5-HT2AR) modulators
Source: PLoS Comput Biol. 2025 Sep 3;21(9):e1013000. doi: 10.1371/journal.pcbi.1013000 (PMC12443254; doi:10.1371/journal.pcbi.1013000)

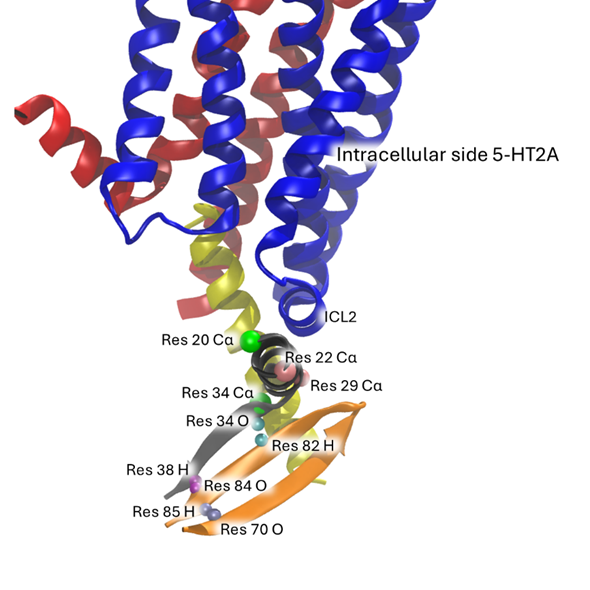

Supplement: S1 Fig — Intracellular side of 5-HT2A (blue and red) with the G protein construct (yellow, silver and orange). For each atom pair to restrain a color was assigned for easy referencing in S1 Table. (TIF) [file pcbi.1013000.s004.tif]

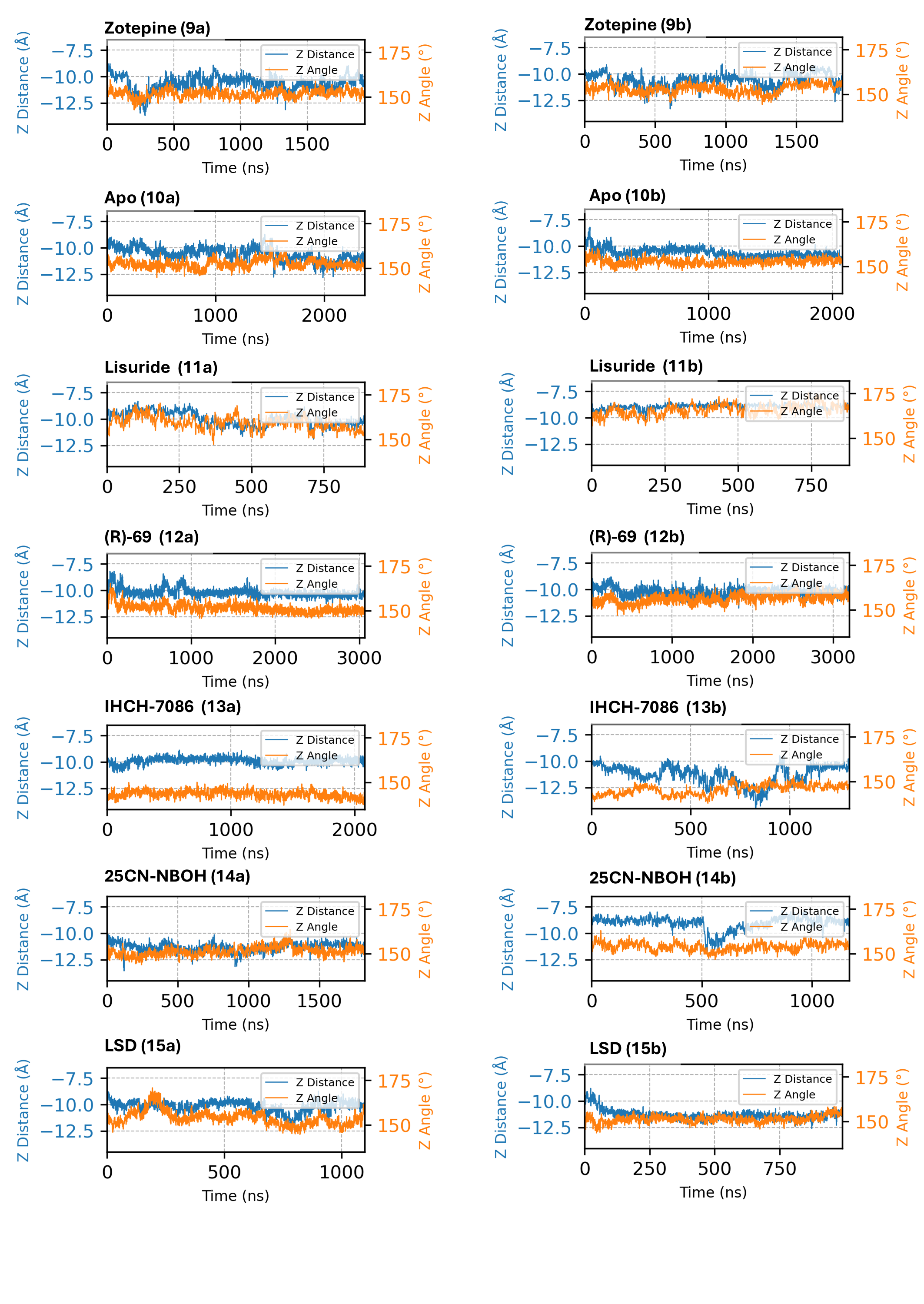

Supplement: S2 Fig — The Z-distance is defined as the difference in the z-coordinate between the centroid of TM2, TM3, TM6, and TM7 (as defined in S2 Table) and the centroid of residues 238–244 of the G protein construct. The Z-angle is the out-of-plane angle between the xy-plane and the vector defined by the centroid of residues 238–244 and the centroid of residues 232–238. (TIF) [file pcbi.1013000.s005.tif]

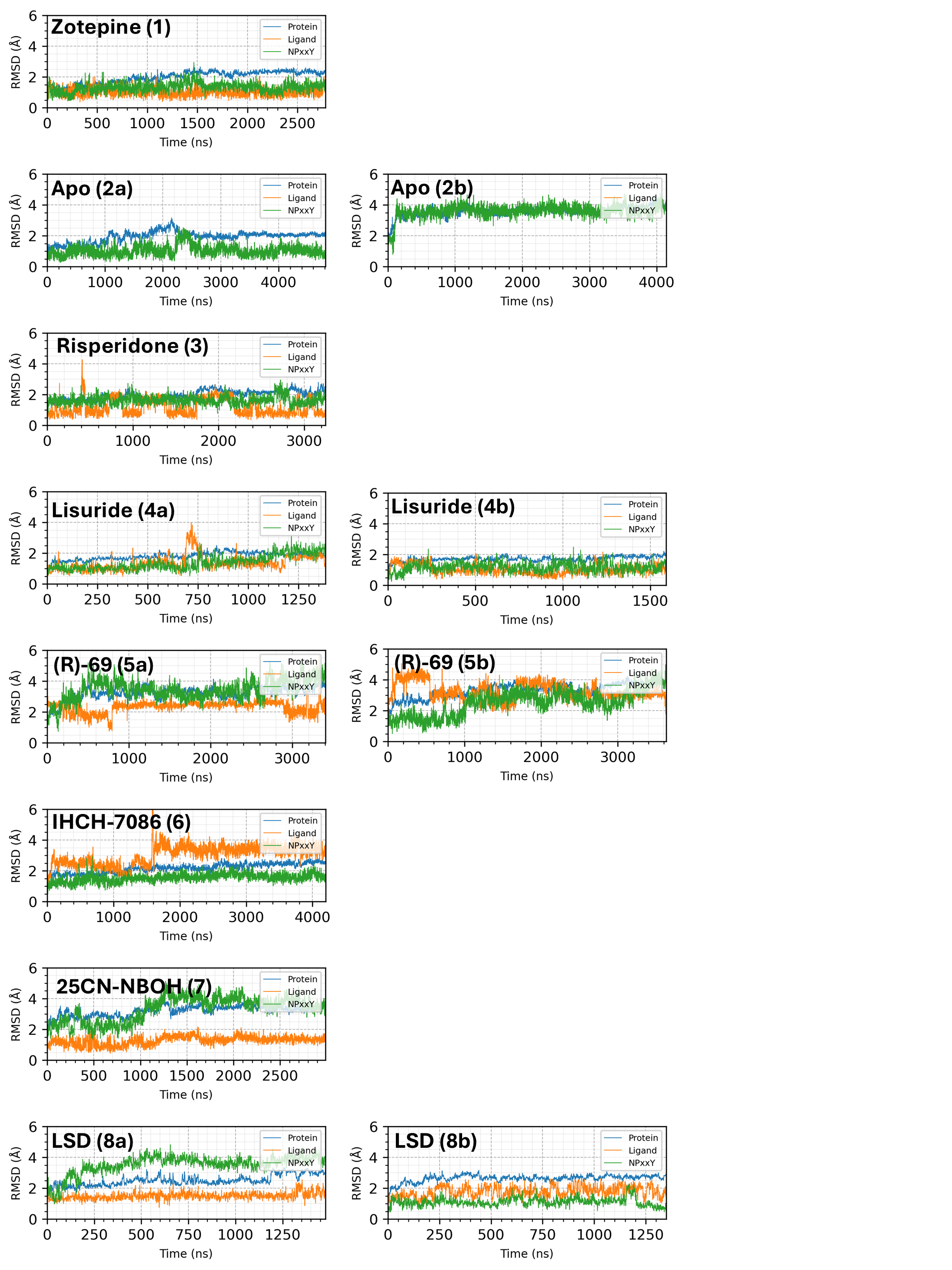

Supplement: S3 Fig — (TIF) [file pcbi.1013000.s006.tif]

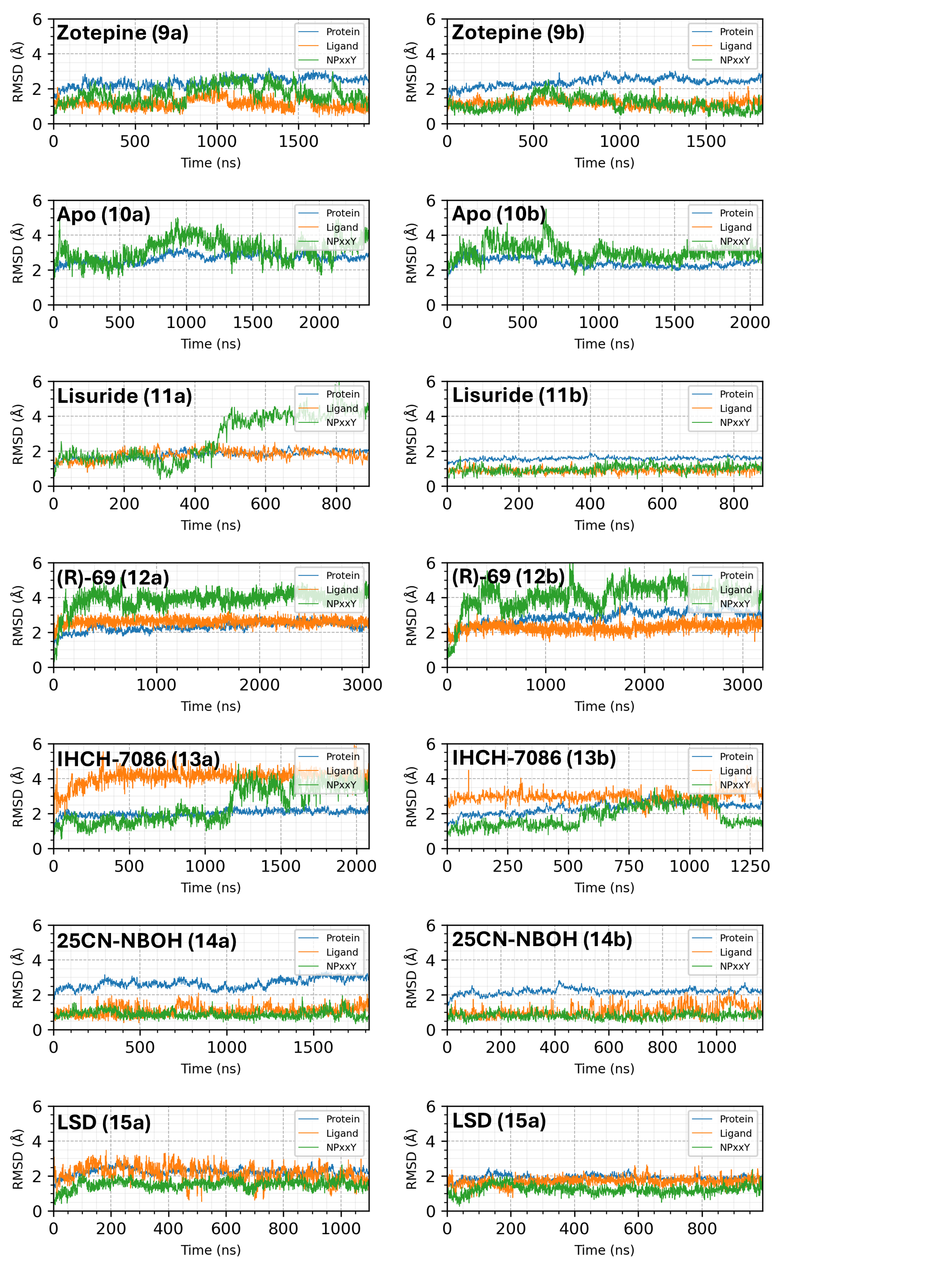

Supplement: S4 Fig — (TIF) [file pcbi.1013000.s007.tif]

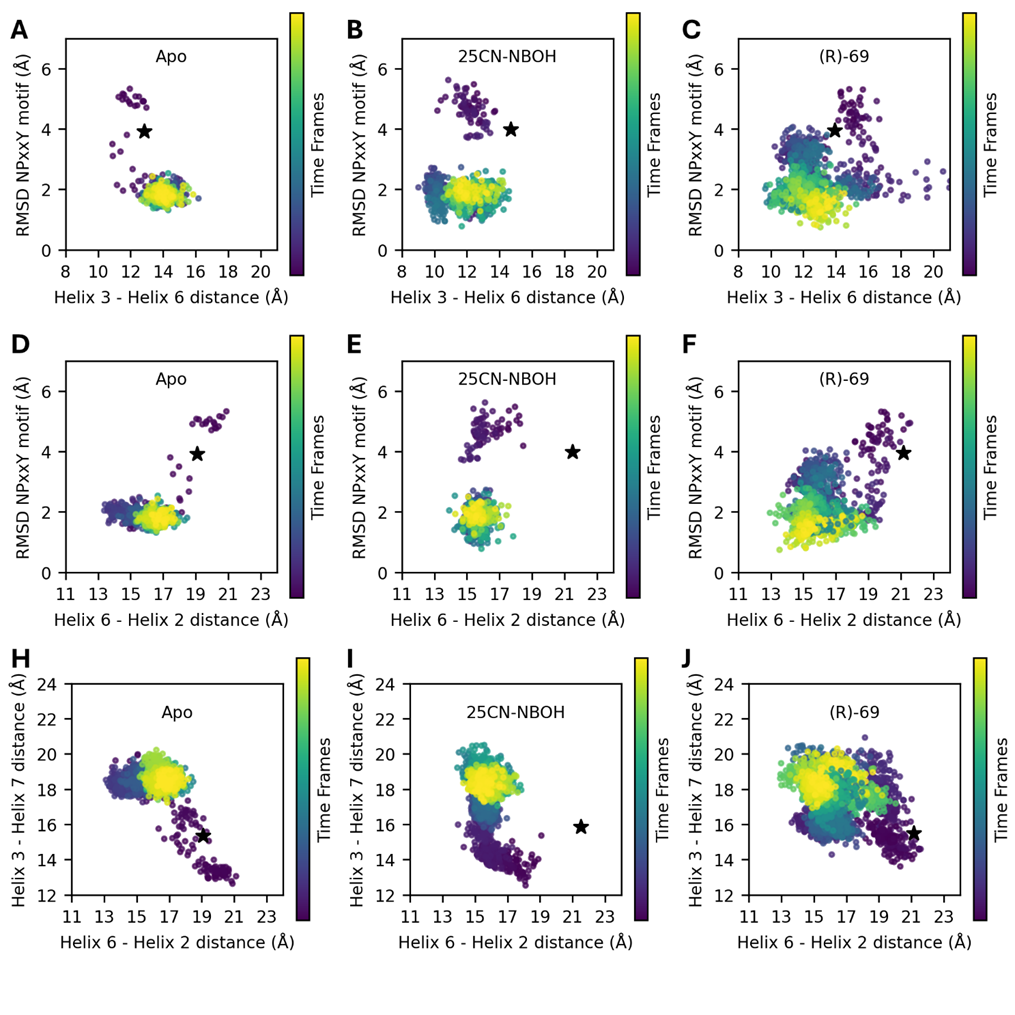

Supplement: S5 Fig — Time series for simulations 2b, 5 and 6 represented in (A-C) by the distance TM3–6 and the RMSD of the NPxxY motif relative to the inactive state; in (D-F) by the distance TM2–6 and the RMSD of the NPxxY motif and in (H-I) by the distances TM2–6 and TM3–7. (TIF) [file pcbi.1013000.s008.tif]

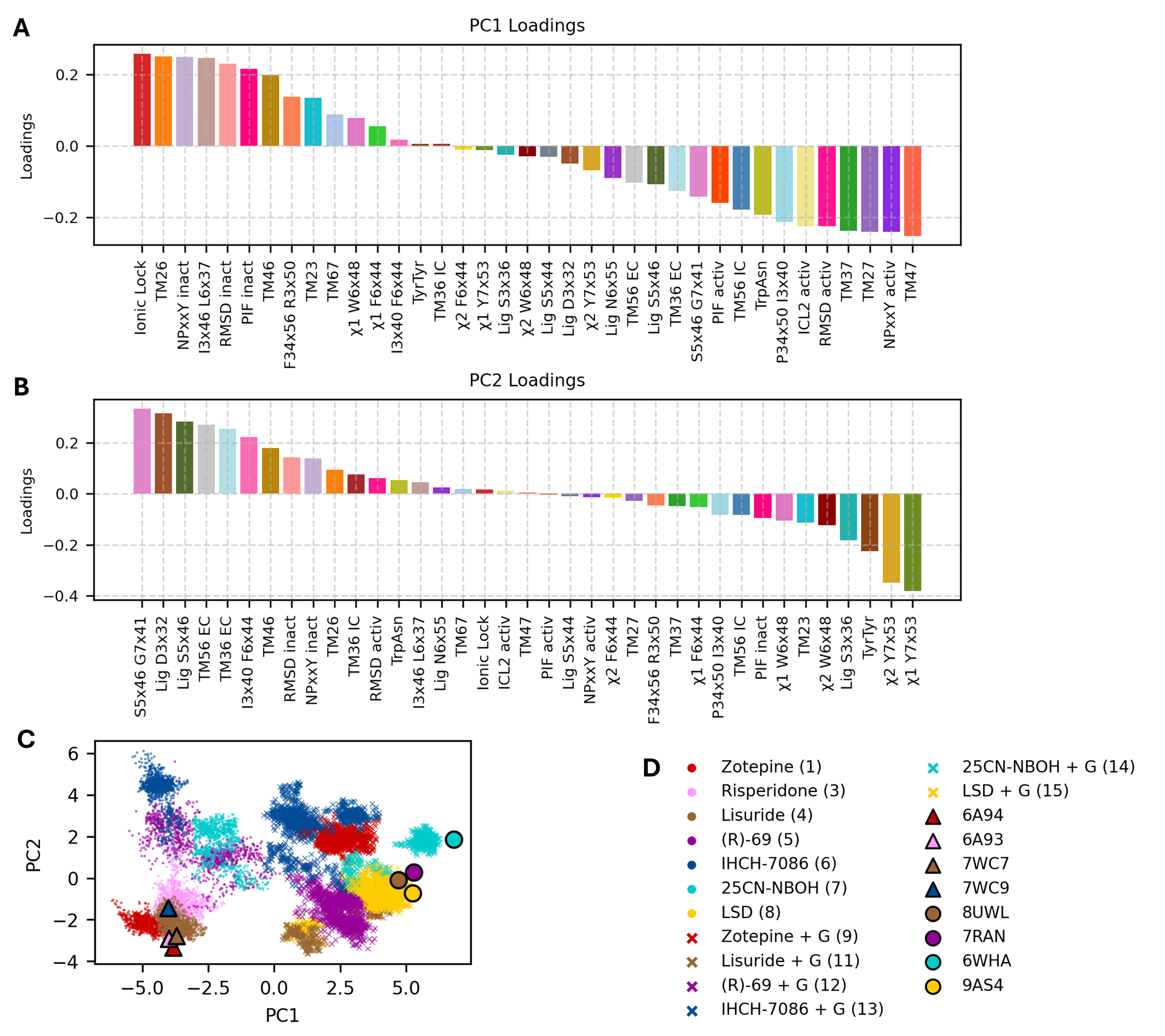

Supplement: S6 Fig — The eigenvalues of the covariance matrix are 11.9 and 4.3, respectively, with explained variance ratios of 32% and 12% for PC1 and PC2. (C) Projection of simulations 1–15 and corresponding X-ray/cryo-EM structures onto the first two principal components. (TIF) [file pcbi.1013000.s009.tif]

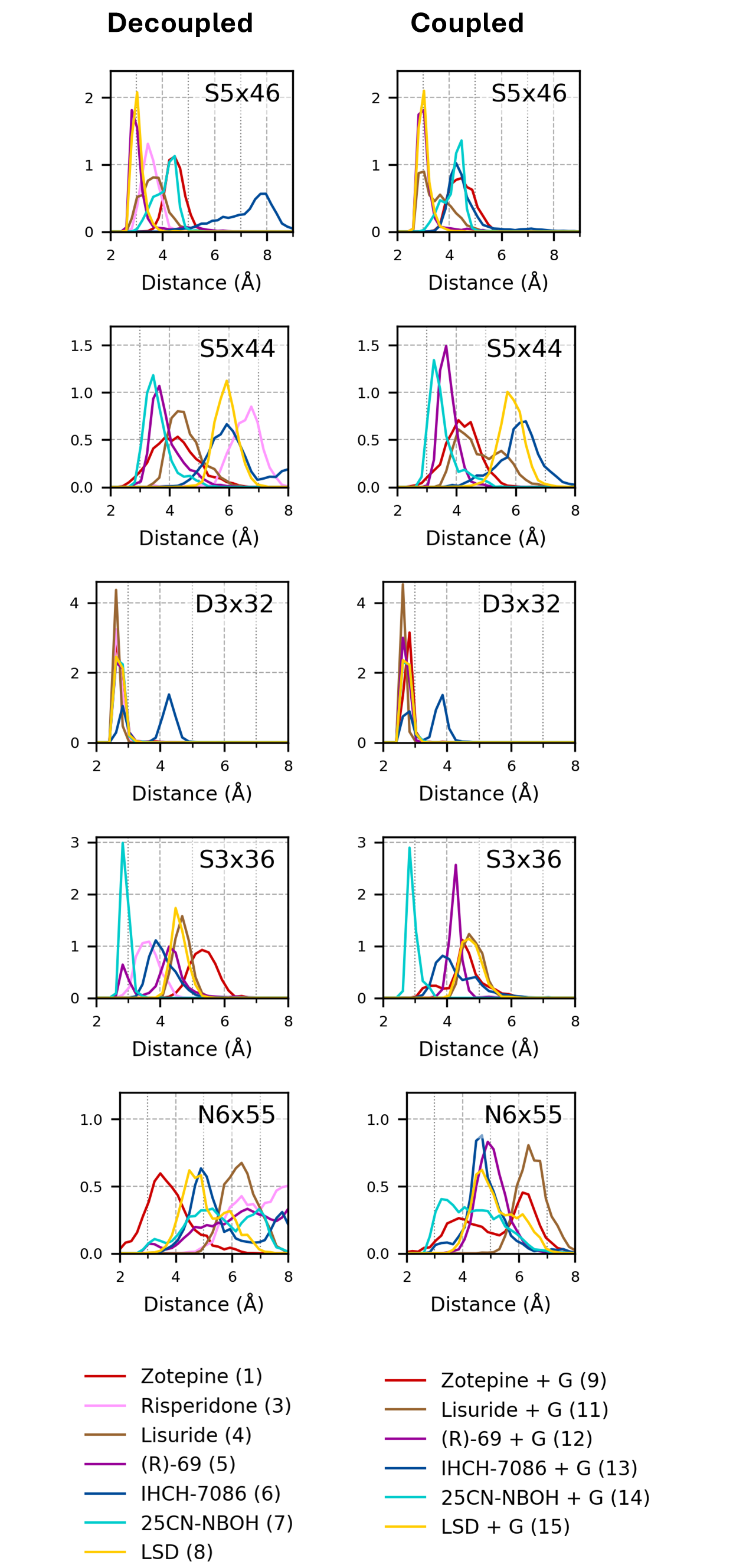

Supplement: S7 Fig — Distance distributions between the closest polar non-hydrogen atom of the ligand and residues S5x46, S5x44, D3x32, S3x36, N6x55. (TIF) [file pcbi.1013000.s010.tif]
