# Supplementary material for: Molecular dynamics study of differential effects of serotonin-2A-receptor (5-HT2AR) modulators
Source: PLoS Comput Biol. 2025 Sep 3;21(9):e1013000. doi: 10.1371/journal.pcbi.1013000 (PMC12443254; doi:10.1371/journal.pcbi.1013000)
Supplement: Attachment — Submitted filename: response-to-reviewers.pdf [file pcbi.1013000.s012.pdf]

Reviewers' comments:

Reviewer's Responses to Questions

Reviewer #1*: The present article by Peeters et al questions the role of hallucinations in the antidepressant-like effect of 5-HT2A receptor agonists and the respective contribution of separate signals associated with G protein Gq and β-arrestin binding. This work is part of a concept suggesting that partial agonists with low efficacies can give rise to antidepressant effects without psychedelic properties regardless of their activation pathway. However, this needs to be confirmed by new studies, on different scales, and it is in this context that this work takes place. One of the main messages delivered herein is that "hallucinations result from excessive populations of activated 5-HT2A and that a non-hallucinogenic antidepressant effect could be achieved by a sufficiently mild activating ligand. This may imply an energetic effect that weakly favors the "fully activated".*
The work and hypotheses raised are interesting and paves the way for a fascinating debate. My (non-specialist) questions concern more integrated concepts, considering the fact that these 5-HT2A receptor ligands are injected in living individuals. I encourage authors to consider them to enrich their discussion.

1) This study focuses on the 5-HT2A receptor but the pharmacological ligand currently available and intended for in vivo administration (either in humans or in animals) also bind and activate other serotonergic (e.g. 5-HT2B/2C – 5-HT1A receptor type) and non-serotonergic receptors (e.g. TrkB). To what extent these elements participate in reinforcing, or on the contrary, attenuating the 5-HT2A receptor signalling pathways should be taken into account and discussed here.

We appreciate the reviewer's suggestion to emphasize this pharmacological complexity. We now dedicated a paragraph in the introduction to the involvement of other serotonergic and non-serotonergic systems, such as 5-HT1A, TrkB, and potentially others. In addition, we mentioned it at the end of the conclusions as a potential follow-up line of research.

2) Can we imagine genetic mutation in specific domain of the 5-HT2A receptor in human that could contribute to modify the pscychopharmacological activity of 5-HT2A receptors in terms of hallucinogenic and/or antidepressant effects? Is there any evidence to support this view in humans?

We thank the reviewer for bringing up this interesting point. While it is somewhat outside the scope of the present manuscript, it would indeed seem attractive to computationally study point mutations that are associated with altered receptor function. However, in the specific case of 5-HT2A, the most prominent instances of such mutations turn out to be impractical to study by MD. Specifically, the rs7333412 single nucleotide polymorphism (SNP), located in the 3' untranslated region and the second intron of *HTR2A*, has been associated with changes in serotonin transporter binding potential (Laje2010) and poor antidepressant response (Qesseveur2016). Unfortunately, our MD techniques only allow to simulate variations in the protein's actual primary structure, not in its expression. A second well-known mutation is the missense variant rs6313 (His452Tyr), which has been implicated in various psychiatric

phenotypes, including severe depression (Petit et al., 2014), mood disorder polarity (Pantovic-Stefanovic et al., 2024), and antidepressant-induced dysphoria in bipolar disorder (Fortinguerra et al., 2019). Functional studies suggest that the His452Tyr substitution can alter 5-HT2A receptor signaling (Hazelwood et al., 2004). However, this residue is located near the intracellular C-terminal tail—a region that is unresolved in current structural models and therefore likely to be disordered (at least in the absence of intracellular binding partners that have not been included in X-ray crystallography and cryo-EM experiments). Accordingly, we speculate that the His452Tyr mutation primarily affects the receptor's selectivity with respect to intracellular G protein and/or beta arrestin pathways, rather than reshaping the receptor's conformational landscape. This makes it particularly challenging to study this domain by MD, let alone to obtain relevant conclusions out of doing so. In summary, it would in principle seem attractive to study point mutations with MD techniques in a future study, on the condition that mutations can be identified that are both eligible to be studied this way and of sufficient interest to the community.

3) Minor points
Line49 : it would be interesting to provide here the affinity of these compounds towards the 5-HT2AR, both in human and rodent.

With this comment, the reviewer is touching upon a major challenge in the interpretation of our results: affinity and activity figures obtained by different sources using different assay conditions (radioligand used, cell line, ...) varied up to an order of magnitude and even relative values were often only qualitatively consistent. It are these qualitative trends that we leverage in our discussion; in the context of the present MD study, we feel uncomfortable choosing one reference over another for the purpose of tabulating quantitative experimental results. Moreover, the utility of doing so is limited by the fact that our MD study itself is largely qualitative/descriptive in nature. Finally, there is a distinction between affinity (how well does a ligand bind to the receptor) and the different forms of activity (how much G-protein signalling / beta arrestin signaling is produced by a give dose / at which dose are there observable hallucinogenic / antidepressant effects?) In most experimental setups, the latter data would need to be corrected for differences in affinity in order to be directly comparable to our simulations, where we assume that the ligand **is already bound** and try to correlate ensuing conformational changes in the receptor with different effects.

Line 51: replace the term “pharmaceutical activation” by “pharmacological activation”

- Thank you for pointing out this error. We have corrected 'pharmaceutical activation' to 'pharmacological activation'.

Reviewer #2: Summary
In this work, Peeters et al. describe a molecular dynamics study of the 5-HT2A receptor in complexes with ligands with diverse pharmacological profiles, ranging from the inverse agonist ritanserin, neutral antagonist zotepine to partial and full agonists 25CN-NBOH, IHCH-7086, and

(R)-69, aiming to find structural features that could be linked to distinct pharmacological profiles. Using PCA analysis on a series of receptor descriptors, the authors highlight important features and descriptors potentially associated with the distinct ligand modalities. The authors also identify a pair of intracellular distances as key descriptors for the modality and pharmacological profiles of compounds.
Despite the relevance of such a study in the context of the renaissance of psychedelics as potential pharmacotherapies, the poor preparation of model systems, lack of simulation replicas, lack of statistical analysis, and absence of control/validation modeling experiments jeopardize the manuscript's results and conclusions. These methodological concerns need to be addressed by the authors before this manuscript can be considered for publication in PLOS Computational Biology.

Major concerns
- The computational experiments are poorly designed. Little attention was given to the preparation of proteins. The experimental 5-HT2A structures have different sequence lengths and different missing domains (including loops and portions of TMs, like the missing TM1 in PDBs 6WHA and 7RAN). It is not clear from the manuscript how these differences were addressed to make the results comparable across simulations and ligand-receptor complexes. Have optimized models been built, ensuring that the receptor have the same sequence length for all systems? Else, how can the results be comparable?

- We are grateful for the reviewer's concerns regarding the system preparation. It made us realize that the methods section on the system preparation lacked clarity, giving the impression that the systems were not carefully prepared. We reworked this part and everything should be clear now.
- We acknowledge that the (long) N- and C-terminus differ slightly between the experimental structures. We did not model the missing residues at the termini, with the exception of the C-terminus of 6WHA, where the helix was too short. In this case, residues 394–401 were modeled using CHARMM-GUI pdb manipulator so this so the C-terminus was of comparable length as the other templates. During our simulations, these termini mainly interacted with the solvent. We believe that the minor differences in sequence length at the termini have a minimal impact on the overall receptor behavior and do not significantly affect the comparability of our results. Importantly, the transmembrane domains were consistently modeled across all systems (see answers next questions).

- Where were the long N- and C-termini truncated? More importantly, what treatment was given to the missing ECL3? Has it been left truncated/open, or has it been closed via some sort of pseudo-loop? These different treatments for the ECL3 can strongly affect the intracellular distances between TMs, from which most of the conclusions are derived, and yet, this is not properly described and apparently this treatment if left to an automated tool to address (Charmm GUI's PDB manipulator) with little control over the output and admittedly a "rudimentary" tool (sic. L138).

- The (long) N-terminus started to be resolved in the experimental structures at residue 69 for 6A93 and 6A94 and residue 71 in the case of 7WC9. For 6WHA and 7RAN we copied missing residues 71 to 83 and 71 to 79 respectively from template 7WC9 after aligning it carefully so there was a good match.
- The (long) C-terminus was resolved up to residues 399, 399, 399, 393 and 397 for 6A93, 6A94, 7WC9, 6WHA and 7RAN, respectively. In the case of 6WHA we employed Charmm-GUI to model the remaining residues (394 to 401) of helix 8 so it had a comparable length as the other PDBs.
- The remaining missing residues of 6WHA (with the exception of ECL3) were modelled as well with Charmm-GUI, though this is a rudimentary tool, it served well with filling in the missing residues. These were manually inspected and no irregularities were observed.
- Charmm-GUI's modelling tool failed to model the missing residues of ECL3 (348-353) for 6WHA. These coordinates were copied from PDB-template 7WC9 after carefully aligned the surrounding secondary structure elements with respect to 6WHA. ECL3 contains a disulfide bond between residues Cys349 and Cys353 which was patched during system preparation. After the minimization and heating steps ECL3 was manually checked, more specifically it was inspected that the chiral centers remained correct after the minimization step.
- This information is now included in the methods section (see also the previous comment).

*- It is also not correct to "copy" the coordinates from TM1 from an inactive state structure (PDB 7WC9) into the active state receptor. A proper homology modeling tool (e.g., Modeller, Swiss-Model, etc.) should have been used for template-based modeling of the missing TM, which would take into account the neighboring atoms when building the side chains, with interactions and repulsion properly addressed via molecular mechanism and a force fie*ld.

In our experience, using homology modeling software for loops requires substantial effort and diligence to get robust and reliable results. In the present study, it is also unnecessary, because we are working with largely **identical** (rather than homologous) sequences and because there is always a template to be found with a nearly-identical conformation in the vicinity of the missing loop. In this light, we preferred to simply copy an experimental loop conformation rather than attempting to cajole a homology modeling program into producing what would ideally be the exact same result. As part of the subsequent CHARMM-GUI workflow, the system was carefully relaxed and pre-equilibrated, then allowed to further equilibrate in the initial stages of our (long) MD runs; we feel this should be sufficient to "clean up" imperfections in the copied loop conformations.

- What treatment was given to the sodium binding site in the vicinity of D120$^{2.50}$? Has this residue's protonation state been properly adjusted to reflect the receptor state? (See https://pubs.acs.org/doi/full/10.1021/acs.jcim.4c01125).

Asp120 was left in its "default" deprotonated state. The reference cited by the reviewer found a pKa difference between the active and inactive state for A2AR and B2AR but not for CB1R, NT1R, and GHSR. We did not find conclusive evidence that this mechanism is relevant for the 5-HT2AR and examining this through computational means is beyond the scope of the present study. However, the conformational transitions we observed are remarkably consistent, which seems to suggest that a hypothetical protonation of Asp120 and unbinding of sodium upon activation may modulate the kinetics of activation and/or the equilibrium between the receptor's active and inactive states, but not sufficiently to reverse a strong conformational preference. Accordingly, if we were to attempt to *quantify* the aforementioned equilibrium in future work, a closer examination of Asp120 would seem worthwhile.

- Why include only arbitrary parts of the mini-G protein? This apparently resulted in such immense artifacts and unrealistic G protein-coupled complexes that the atoms had to be kept in place via positional restraints during the simulations. How can these results be trusted? Indeed, the experimental mini-Gαq proteins in PDBs 6WHA and 7RAN are Gq/Gi/Gs-chimeras, but these mutations could be reverted with a template-based homology modeling. Also, there are templates available with the natural (non-chimera) mini-Gαq-subunit and even with the complete Gαq subunit. However, if the authors had chosen to build an AlphaFold Multimer model of the Gq-coupled 5-HT2A, (see https://www.biorxiv.org/content/10.1101/2021.10.04.463034v2, available and open source since 2022), it would be less artificial than the method applied in this manuscript. Has this G protein-restrained construct been validated in comparison to the non-truncated mini-G protein?

- We are well aware that plenty of mini-G structural data is available; indeed, if we had blindly submitted the cryo-EM structures to CHARMM-GUI, we would have had a simulation system containing the same mini-G construct as in the experiment. The reason why we chose not to is a computational one. Indeed, including the full mini-Gαq subunit would roughly double the required computational resources because it extends far into the solvent, thus doubling the Z dimension of the simulation box. Engineering a construct that retains the interactions with the 5-HT2AR while being far lighter to simulate allowed us to obtain roughly twice as much sampling within our computational and practical constraints. Since sampling is the main challenge in this study (as discussed at length in response to another comment below), we feel this is a favorable trade-off.
- The parts of the mini-Gαq protein we included are not at all arbitrary; they were chosen based on their proximity to the receptor while not excessively disrupting secondary structure elements. Also, the atoms are not kept in place with positional restraints but with distance restraints; this distinction is important because it allows the construct to freely rotate and translate. Restraints are generally necessary for the remaining secondary structure elements to retain their relative positions and thus realistically mimic the full protein. Indeed, introducing restraints is common practice when engineering this type of construct and we did not attempt to simulate it without; doing so would make little sense because the construct would be able to adapt itself to the receptor and membrane in ways the full Gαq protein cannot. That said, we did perform

trial runs with different restraint strengths in order to obtain a realistic degree of adaptation (neither too rigid nor too floppy) based on the different available structures as well as experience with protein simulations.

- There is no analysis of MD simulation convergence. The RMSD of the receptor (all residues and residues in the 7-TM domain), of this G protein "construct", and especially the RMSD of ligands should be shown. Are the ligands still bound to the receptor at the end of the simulations?

We added the RMSD plots in de SI. However, the G protein construct was able to perform rotational motions in the xz-plane. While these rotations were limited in amplitude in both directions, they gave rise to superficially high RMSD values and fluctuations that would mask any significant effects (when centering the system on the receptor, but for the present purpose, it would make little sense to do otherwise). In order to obtain more relevant data, we plotted time series of the z-distance between the interacting helix of the G protein and the receptor and of the angle between this helix and the xy-plane. These quantities show that the G protein construct remains stabley bound during the MD simulation. Additionally, we observe that ICL2 assumes a helical geometry in de G protein bound simulations which relates to an active state. In the simulations where the G protein was removed, ICL2 rapidly changed to a disordered loop, a feature belonging to the inactive state. This indicates that our construct helps stabilize active state features in the same fashion as the full protein.

- The MD simulations are inconsistent. Why do the simulations have different lengths? This is not explained in the manuscript. How are frames selected/sampled for the analysis? Hopefully, the same number of frames and the same frame selection criteria are applied for all simulation systems (despite different simulation lengths), or else the analyses would be biased to the systems with more frames.

- We followed the reviewer's suggestion and the PCA is now performed on the same number of frames for each simulation. It should be noted that this did not significantly change the results, as PCA is relatively robust with respect to the sampling densities (data not shown; the PCA in the final manuscript also includes the new simulations, which did make a small but significant difference in PC2).
- The simulations are of different lengths due to computational constraints; see the next point.

- All simulations should have the same length and be performed in triplicate, using different random seeds for the initial velocities, to increase the statistical significance of observations and to assess the robustness and reproducibility of the findings. Use the RMSD plots to (consistently) remove from your analysis the initial frames in which the systems are not equilibrated.

- *We resp*ectfully disagree. Simulating 3 x 1 microsecond has no advantage over simulating 1 x 3 microseconds. In fact, running shorter simulations in parallel has a

disadvantage: the odds of observing transitions that are very slow decrease faster than linear with shorter simulation time. Where "very slow" includes large-scale transitions that happen over a relatively short time (in the 10 ns order of magnitude) scale but require a cascade of small-scale transitions (such as the toggling of microswitches in a certain order) to occur first.

The reason why it has become somewhat common to run multiple parallel instances of a simulation is not statistical but computational: if one has unlimited computational resources available, a single simulation will parallelize poorly, and running multiple simulations in parallel will yield the same amount of sampling (i.e. the same total number of microseconds) in less "wall time". Unfortunately, we are a small computational group based in Belgium and we do not in fact have unlimited computational resources at our disposal. Therefore, we absorbed the operational penalty of having to wait several weeks for the results of our non-duplicated simulations, with the advantage of having higher odds to observe rare-and-very-slow transitions.

To assess the robustness as intended by the reviewer, one could divide our trajectories into multiple parts and compare averaged properties calculated on the parts to ascertain how well the averages are converged, as is common in single-trajectory MD studies that are focused on calculating such averaged properties. However, the focus of the present study lies not on calculating averages but on mapping states and observing transitions. These transitions did indeed turn out to be rare and very slow with respect to our simulation time scales (see RMSD timeseries; Figures S3 and S4); average properties that are relevant to the transition will obviously differ substantially between partial trajectories before and after the transition, and the trivial conclusion would be that these properties are not converged. However, allowing them to converge is neither the point of this study nor computationally feasible with plain MD. Proteins undergo different transitions at different time scales, and until we can simulate for several hundreds of *milli*seconds, there will always be transitions that are rare and very slow with respect to feasible simulation times. We feel that reporting such "cutting-edge" transitions is scientifically valuable, as long as they are interpreted with sufficient care. Specifically, we deliberately make no quantitative claims about averaged properties, convergence or kinetics; rather, we qualitatively describe the transitions we observe and carefully formulate tentative links with known biological properties of the ligands we studied. Future experiments (or computations with far more resources) may confirm or refute these hypotheses; this does not imply that we have made no contribution to the advancement of science.

- Similarly, the trajectories are different lengths because of computational constraints. This may make it problematic to compare averaged properties between trajectories, but again, we are not doing that. Even so, the reviewer is fundamentally correct that the different lengths are a potential source of bias. We were acutely aware of this when drafting the manuscript and have been careful to avoid drawing firm conclusions that might be biased in this fashion. Scientists have been doing so since the early days of protein MD, even if the difficulty of interpreting results that involve rare transitions has since shifted to longer time scales and rarer events.

- Removing the non-equilibrated part of a trajectory is indeed good practice for calculating averages. However, it should be noted that, especially in the context of protein simulations, “equilibrated” is only defined with respect to the properties one is interested in. In our study, there are several instances where we deliberately perturbed an experimental structure (e.g. by removing the G protein construct) to observe the transition the system would undergo in response. In this context, it would not make sense to eliminate the part of the trajectory before the transition. Not only would it be hard to describe a transition without two end points, but we expect that most of the conformational transitions we observe are in fact equilibria at longer (multi-*milli*second) time scales, e.g. that a 5-HT2A receptor with an activating ligand but no G protein would be in a (partially) activated state for a (very small but kinetically significant) fraction of the time. For the purpose of calculating the associated equilibrium constant in a “brute force” fashion, the system would only be truly converged when many back-and-forth transitions are observed. This is trivially out of computational reach, which is why we make no statements about equilibrium constants. However, since there is a fair chance that the pre-transition geometry is part of the equilibrium, we describe it as such. While doing so, we do eliminate the part of the trajectory where we see immediate adaptations of the (low resolution) experimental structure to the (significantly different) simulation environment, because that part is indeed not equilibrated for our purpose. Table S3 lists the discarded frames for each simulation that were not "equilibrated" for our purposes.
- Similarly, when employing PCA analysis for the purpose we are using it in our manuscript, not including the part of the trajectory before the transition would compromise its ability to comprehensively map accessible conformations and identify correlated components associated with transitions. This has now been addressed in the material and methods section.

- Why use 5 different PDB templates that introduce uncertainty and variability to the starting models (due to their different sequences, crystallography artifacts, etc.), while they actually represent only two receptor states – the active/G protein-coupled state and the inactive – just look at their RMSD to one another. Instead, it would be more valuable to focus on just two properly prepared models for the active and inactive states, and then cross-dock and simulate the distinct ligands using these two starting references. In the current manuscript, it is hard to discriminate the effects of the ligands from the effects of the various receptor templates. For example, PDB 7WC9 has a partial agonist bound, but the receptor is in the inactive state due to the Bril-fusion. What does this receptor template mean in the activation energy surface? Probably nothing, it is a crystallography artifact, so why include this?

- A large part of our endeavor was to map the conformational space of the receptor, with various ligands and in the presence and absence of the G protein, investigating the “biased agonism” hypothesis in the process. Starting from only two templates would run contrary to these goals and would itself be an unacceptable source of bias (“knowing that biased agonism of 5-HT2A has been suggested in the literature, why start only from a single inactive and a single active conformation?”)

- Furthermore, docking is notoriously unreliable and it would be extremely non-trivial to capture all the adaptations of the protein in response to the ligand, while these adaptations are readily available in the experimental structures. For instance, the importance of side chain conformations in the activation mechanism is well-documented (and differences at this level do not automatically result in high RMSDs of the whole protein).
- That said, for some of the specific initial states we wished to simulate, no experimental structure was available, in which case we did resort to the reviewer's suggested approach. Specifically, cross-docking was performed for the G protein simulations with Zotepine, IHCH-7086 and 25CN-NBOH. The results are consistent with the G protein simulation with (R)-69 as well as the newly performed simulations with lisuride and LSD (for which cryo-EM structures were available).
- For the specific example of 7WC9, we would like to point out that one of the major findings of our study is that an activating ligand alone is insufficient for a full transition to an activated state – that it merely modulates the energy surface such that the transition to the active state upon binding the G protein is facilitated, in line with Reference 39 (Dror et al., https://doi.org/10.1073/pnas.1110499108). In this case, an inactive state with a partial agonist bound is not that artificial at all! Our study shows that the aforementioned modulation results in a ligand-dependent shift in the inter-helix distances within the basin of inactive states, which we feel is an observation worth reporting.
- Finally, we wish to stress that we never claimed to be quantifying the activation energy surface. We have been very explicit about this with the following passage: "(...) the Boltzmann inverted populations obtained from the different MD simulations are shown in Figs 5 and 6. While these should not be interpreted as converged free energy profiles, the logarithm makes the large range of population densities more manageable as well as expressing them in more intuitive units of energy." Overall, we have the impression that the referee is reviewing our qualitative study as if it were a quantitative one.

- PCA is a powerful tool for dimensionality reduction and, via the loading plots, a useful tool to identify the most significant factors contributing to the observed variance. However, just describing the PCA and loading plots does not add to the scientific knowledge about the 5-HT2A receptor. Instead, the authors should use the plots to identify the most significant features and then assess how these features evolve throughout the different simulations – i.e., show the values trajectory and compare them between model systems, applying a statistical test (e.g., ANOVA) to evaluate if the observed differences are statistically significant.

We acknowledge that we initially overinterpreted the PCA loading plots and placed too much emphasis on them. This section has now been rewritten with two main goals. The first was to evaluate whether the features reported in the literature also emerge from our data. The second was to highlight the most prominent DOFs identified by the PCA, which are further explored in subsequent sections. The PCA results showed that movements involving TM26, TM37, and the NPxxY motif contribute most to the variance captured in PC1. These features are now discussed in detail in the following section. We

then focused on the key contributors to PC2, which are now addressed in the subsequent section. The concluding section on large-scale receptor movements integrates these findings, showing how both microswitches and broader conformational changes appear to correlate with the activating potential of the ligands.

- It is important to clarify that the aim of our manuscript is not to develop a predictive model capable of classifying ligands as hallucinogenic or non-hallucinogenic. Rather, the identified pair of interhelical distances (TM3–TM7 and TM2–TM6) was used in a qualitative manner to support our hypothesis: psychedelic compounds, although not always full agonists, tend to strongly activate the receptor by stabilizing conformations that favor coupling to intracellular partners. In contrast, non-psychedelic compounds, while capable of forming complexes with intracellular binding partners, appear to favor less optimal (non-canonical) active-like conformations. The inclusion of Lisuride and LSD strengthens this hypothesis. Given that our approach is not to quantify differences between discrete populations but to qualitatively describe how ligands modulate the overall conformational landscape of the receptor, it is not obvious which exact quantities would need to be subjected to a statistical test (nor what doing so would contribute to the article).

- There is no description or analysis of ligand-protein interaction and how those could impact or be linked to the observed PCA outcomes, or how they vary among the distinct ligand modalities. Perhaps the ligand-receptor interaction could be included among the PCA variables.

We agree with the reviewer's comment that ligand–receptor interactions were not sufficiently considered. To address this, we have added an additional PCA analysis incorporating five key polar interactions. This analysis provided valuable insights, and a new section discussing ligand interactions has been added to the manuscript.

- The manuscript is confusing and poorly written (please, check spelling and grammar). Imprecise and unscientific language is frequently used. For example: "left out" L142, "by means of trial and error" L154, "Interpretations… were performed in VMD" L194, "after some equilibration" L435, "Therefore, the question becomes more academic" L488.

We carefully inspected the manuscript for imprecise or unscientific language and rephrased it wherever possible.

- The eigenvalues of the correlation matrices are not shown.

These values have now been added.

- P220: It is not correct to conclude, based on the PCA of distances, that the residue motions are correlated. The correct approach is dynamic cross-correlation or covariance analysis over the trajectories, not an arbitrary output metric.

We thank the reviewer for pointing out his instance of inaccurate wording. We did not actually intend to speak about motion specifically, so this has been rephrased.

- L213: What is the relevance of having the PCA performed on all the trajectories versus the PCA of the coupled and non-coupled receptor? This is not discussed further in the manuscript, and, ultimately, the same data is being analyzed twice.

We are grateful that the reviewer brought this to our attention. The separate PCAs of the coupled and uncoupled systems were essentially a remnant from an earlier stage of this project; we recognize that they do not contribute much to the present manuscript and have therefore removed them. Conversely, a second PCA that includes ligand–receptor interactions (and therefore necessarily excludes the apo simulations) has been added in response to another comment (see above).

- L221-223: There is no reference to TM5 in any of the graphs in Figure 2A-C, and nothing in these figures supports the conclusion that “relevant active and inactive states were sampled”. How is this being assessed?

This was mainly derived from the RMSD related DOFs of the (in)active state(s). This has now been properly addressed in the manuscript.

- L242: It is not possible to assess or conclude via these computational experiments that the "differential nature of the ligands has a direct impact on these degrees of freedom" as these observed differences could simply be a result of the different starting points (distinct initial receptor conformations obtained from the experimental structures and their artifacts) rather than a change promoted by the ligands – as the authors admit in L263 – indicating the complexes are “stuck” around local minima. The study lacks control simulations to isolate the effects of specific ligands or the presence/absence of G proteins, making it harder to directly attribute observed differences to specific factors.

- It is true that simulations initiated from inactive crystal structures tend to remain close to their original experimental conformations. An exception is the zotepine-bound simulation (simulation 1), which—based on the TM3–7 and TM2–6 distances—appears to shift toward an even more "inactive" state. Fortunately, we do observe significant conformational transitions, particularly in simulations where the G protein is present. Additionally, when the G protein is removed from the PDB template, we observe transitions from the active state toward an inactive conformation. In these cases, the receptor adopts a structure that closely resembles the experimentally determined inactive X-ray structures.
- The only conformational transition not observed in our study is from an inactive to an active state. Based on the previous work by Dror et al. (2011), we believe that this transition is extremely rare compared to MD time scales and/or requires the simultaneous binding of a G protein—placing it beyond the reach of standard MD simulations.

- Apart from that "missing transition", we do not understand how the reviewer's "control simulations to isolate the effects of specific ligands or the presence/absence of G proteins" differ from what we did. If the reviewer means "longer simulations", we wish to point out that there are constraints on the computer power we have at our disposal (see above). If the reviewer means "simulations with cross-docked ligands", we do not believe that would be better or more reliable, as pointed out above.
- We included two extra simulations which further supports the hypothesis with regard to mild and strong activating ligands.

- L280: The plots for these systems in the simulations with the G protein-coupled complexes should also be shown and then compared to the "decoupled" systems.

We moved a part of these plots in the main text and added the TM26-NPxxY plots of the G protein-coupled systems.

- L368: The bulge in TM5 is an intrinsic feature of the 5-HT2A receptor encoded in the sequence (look at the sequence alignment of the 5-HT receptor family), which possesses a π-helix caused by the extra residue S242 – therefore, it is visible in all experimental structures, independent of the bound ligand or receptor state. It is worrisome to read that the bulge "disappeared" in your simulation (L371). This suggests a poorly prepared system. Please, reevaluate your computational experiments.

We thank the reviewer for highlighting this concerning phrasing. We intended to explain that the "bulge" is less pronounced. Cao et al. (2022) designed IHCH-7086 specifically for this purpose. This compound lacks the ability to form a hydrogen bond with S242 in an attempt to synthesize a beta-arrestin-biased ligand. As a consequence, F5x47 and F5x48 adopt an alternative conformation, disrupting the pi-pi stacking and making the bulge less pronounced. We have added a section on the TM5 bulge that explains in greater detail how our ligands interact with this structural feature and how they might influence downstream signaling.

- Figure 5 shows that the density profiles of "zotepine + G" and "25CN-NBOH + G" are very similar despite the opposite pharmacological profile of these compounds. From this, the authors conclude, without experimental support, that zotepine locks the receptor in both the active and inactive conformation, i.e., hinders the conformational freedom of the receptor regardless of the initial state. However, one could argue instead that the shift towards the inactive state observed for the other G protein-coupled system (Apo, IHCH-7086, (R)-69) might reflect the unbinding of the G protein construct or artifacts caused by this unnatural construct – this should be properly assess before any conclusion could be drawn from these simulations.

- The passages the reviewer is referring to started with: "While this may easily be a shortcoming of the computational time constraints, an alternative explanation would be that (...)" and further down: "This may simply be a consequence of insufficient sampling, but if not, the simulation results suggest that (...)". It appears that in both instances, the reviewer took the subsequent text out of context. We did not at all

"conclude" that zotepine locks the receptor in both the active and inactive conformation; on the contrary, we started with saying that it was likely an experimental artefact, but *if not*, it could *alternatively* mean that (...) To further emphasize this tentative nature, we removed the second instance and rephrased the first.

- The unbinding of the G protein does not happen in any of the simulations. Timeseries were added displaying the distance between the G protein and the receptor as well as the angle the interacting part of the G protein makes with the receptor. No irregularities were found.

- Having identified this pair of distances (TM3-7, TM2-6) as key descriptors (and predictors) for the modality and pharmacological profiles of ligands, this should be validated by i) statistical analysis, to ensure these differences are indeed significant; ii) simulating ligands that were not included in the previous analysis (i.e., a set of test ligands) to check if their density profiles would match the expected/predicted profile disclosed in Figures 5 and 6. Some obvious choices would be serotonin (by definition, a full and unbiased agonist), LSD (β-arrestin biased, partial agonist and psychedelic, see: https://doi.org/10.1016/j.cell.2020.08.024), Lisuride (β-arrestin-biased, partial agonist, non-psychedelic, see: https://doi.org/10.3389/fmolb.2023.1233743), Pimavanserin (inverse agonist, see: https://doi.org/10.1021/acs.jmedchem.4c01244), all of which been experimental coordinates available. Other alternative validation compounds could be those published at https://doi.org/10.1038/s41467-025-57956-7

While time and computational constraints did not allow for running and analyzing 3 additional sets of simulations, we did add 2 of the 3 suggested ligands to our simulation set: Lisuride and LSD. The results were largely in line with our original interpretation, thus serving as a validation in addition to being a generally beneficial extension to our study. We therefore thank the reviewer for this valuable suggestion.

- L455-470: None of the presented data supports the claims in this paragraph. There is no evidence that 25CN-NBOH is a psychedelic in humans; it actually produces fewer head-twitch responses (HTR) in mice than other psychedelics (https://doi.org/10.1007/s00213-014-3739-3). Serotonin is not a weak binder (Kd = 1.3 nM, see https://doi.org/10.1016/0006-2952(95)02122-1).

- We rephrased this to "hallucinogen-like effects in mouse models." To the best of our knowledge, there are no known compounds that exhibit psychedelic effects in mice but not in humans, or vice versa. Halberstadt2020 et al (https://doi.org/10.1016/j.neuropharm.2019.107933) showed that the HTR in mouse models is predictor for the psychedelic potential in humans.
- As proposed by the reviewer, we added LSD to our set of simulations, a known psychedelic compound. This extra simulation is in line with our hypothesis regarding to mild- and strong activating ligands.
- In comparative studies between 25CN-NBOH and serotonin, it was shown that serotonin is less efficacious than both 25CN-NBOH and LSD. We revised the phrasing

accordingly and added the appropriate references to support the relative efficacy of 25CN-NBOH and LSD.

L472-495: The presented data and results do not support the conclusions. There is no indication that any of the studied compounds have either hallucinogenic or antidepressant effects in humans, as they have not been in a clinical trial. Associating the observed differences in the probability density plots of a couple of CVs (which have not been assessed for convergence and adequate sampling of the phase space) with behavioral effects is overstretching and overinterpretation of the computational results. The alleged equivalence of a small ("microdosing") of a full agonist to the effect of a partial agonist ignores ligand residence time, receptor turnover, receptor desensitization, and signal amplification mechanisms, while also being unrelated to the results discussed in the manuscript.

- Human clinical studies on the exact effects of these compounds have often yielded contradictory results, while results on mouse models seem somewhat more consistent, yet fairly predictive for effects in humans (see previous point). Regardless, we acknowledge that our original phrasing did not clearly reflect the fact that we are mostly using mouse models as a reference, as opposed to human clinical evidence. Accordingly, we appreciate the reviewer pointing this out and have revised the text accordingly to avoid any unintended implications.
- Regarding the interpretation of the computational results, we disagree with the assertion that our data do not support our conclusions. Many of our simulations underwent significant computational transitions, which suggests (1) that we simulated long enough to at least partially overcome starting structure bias and (2) that the state after the transition is likely more favorable than the state before – knowing that the observed transitions are largely consistent between the different ligands. In the end, our simulations revealed distinct ligand-dependent conformational states, which, in our view, supports drawing *cautious, qualitative* links to known behavioral effects—particularly when considered alongside existing literature. Indeed, our findings are consistent with previously reported non-canonical conformations (as cited in Wallach 2023, Dror 2011, and the recent Gumpper 2025 study).
- In response to the reviewer's feedback, we have incorporated additional ligand–receptor DOFs into our analysis and additional simulations with lisuride and LSD. This reinforced our hypothesis that strongly activating agonists stabilize the active conformation, whereas weaker agonists favor non-canonical or partially active states.

Minor comments
- In recent years, the term "psychedelics" has been preferred instead of "hallucinogens". See https://doi.org/10.1055/a-1310-3990

The reviewer brings up a relevant but nontrivial point of debate. The term "hallucinogen" is broader, while "psychedelics" typically refer to partial agonists of the serotonin-2A receptor that give rise to hallucinations. Since the present manuscript is only concerned with 5-HT2A, it would indeed seem logical to only use "psychedelic". However, the involvement of other receptors is often unclear (we dedicated a new paragraph in the introduction to this in response

to a comment by reviewer 1). In this light, it could potentially be argued that at least some of the compounds under consideration should more accurately be characterized as hallucinogens. We did our best to revise the manuscript such that the most appropriate term is used as much as possible, but we do not think it is possible to always pick a term that will please every single potential reader.

- In the introduction, he research question and hypothesis should be made explicit. Currently, the aim of the study does not become fully clear until later in the methods and discussion sections.

We now stated our aims more formally at the end of the introduction.

- How much of the variance can be attributed to each PC? If PC1 and PC2 capture most of the variance, there is no reason to show or mention other PCs, as these might not be relevant.

As stated earlier, we agree with the reviewer's assessment regarding the number of PCs to discuss. We modified the manuscript, limiting ourselves to the first 2 principal components.

- L242: Incorrect figure reference.

This section has been rewritten, and the figure references have been updated accordingly.

- Splitting the manuscript into subsections is unnecessary (or relevant). For example, splitting the introduction into 1.1 and 1.2 sections.

Following the reviewer's suggestion, we have removed the subsections from the introduction. Conversely, we feel that removing the subsections from the discussion would be detrimental to general reader-friendliness.

- P2L47: Those abbreviations need to be spelled out – not obvious to all readers.

This has been addressed in the manuscript.

- L206: What is the meaning of "plethora of microscopic modulations"? This does not make sense.

We rephrased this sentence.

- Since Figure S5 is highly discussed, it should be somehow included in the main document.

A part of S5 is moved into the main text and the "coupled" plots were added for comparison.

- Table 1 should be better organized. For example, grouped into "coupled" and "non-coupled"

complexes, color-coded by the state of the receptor in the initial structure (active vs inactive), with ligands sorted by modality ("pharmacological effect"). A structure with a "None" ligand is called "apo". Spelling and capitalization mistakes should be addressed.

We reworked Table 1 following the reviewer's suggestions.

- In all figures displaying structures, hide the non-polar hydrogens. Increase the contrast between cartoon and residue lines (e.g., color them differently) or consider rendering with another tool like ChimeraX or Pymol rather than VMD.

We appreciate this suggestion and have incorporated it into the manuscript wherever feasible.

- In all figures, add labels to more quickly discriminate between coupled and non-coupled receptor states.

We are grateful for this suggestion and have incorporated it into the manuscript wherever feasible.

- Figure 1: Panel A is not relevant (common knowledge on GPCR activation). Panel B should be enlarged, colors adjusted to increase the contrast of the highlighted key residue sets versus the receptor; and hide nonpolar hydrogens. Instead of showing the microswitches, display the most relevant metrics/distances that are discussed in the manuscript (focused on the features with the most significant loadings from Figure S2). Those are what readers should understand and remember. Improving this figure to show the most relevant DOFs (and only those) can replace Table 1, which is already repeated with further detail in Table S2.

We thank the reviewer for this valuable suggestion. Figure 1 has been revised to focus on the primary degrees of freedom (DOFs) discussed in the manuscript. Key residues are now shown with improved color contrast and nonpolar hydrogens have been hidden. Table 2 is removed and appropriate references to Table S2 and Fig 2 have been added.

Figure 2: Add labels on top of the graphs to more clearly identify the PCA of all systems, coupled and non-coupled receptors. The caption is confusing. Show first the PCA plots and then the loading plots (with arrows).

Figure 2 now displays the PCA results for all trajectories. The loading plots have been separated into individual panels for PC1 and PC2 to improve clarity. Since Table 2 has been removed, this figure now summarizes all DOFs used in the PCA.

- Figure 4: Reorganize the panel to make better use of the page space and increase the figure sizes. Since the χ angles of tryptophan and phenylalanine are discussed heavily across the manuscript, display these in a schematic as they might not be obvious to all readers.

Additional figures displaying these angles have been added to more easily follow the discussion in the main text.

Reviewer #3: This study conducted long-term molecular dynamics simulations of ligand-complexes of the 5-HT2A receptor with antagonists, inverse agonists, G protein-biased agonists, and β-arrestin-biased agonists. The authors analyzed the motion differences in the transmembrane regions and related motifs when the 5-HT2A receptor binds to different types of ligands, contributing to the understanding of the biased activation and antagonism mechanisms of the 5-HT2A receptor. The results should be of help to drug discovery efforts targeting the 5-HT2A receptor.
Suggestions for improving the manuscript are:
1. Further exploration of the interaction differences between related ligands and protein amino acids would be more helpful in elucidating the mechanisms by which different types of ligands influence protein conformation.

We thank the reviewer for this comment. Indeed, further exploration of the ligand-receptor interaction did strengthen the argumentation linking the microscopic modulations with the large scale movements.

2. The article addressed the disconnected regions of the protein (e.g., TM5-6) by capping (without completing the disconnected regions) but did not discuss whether the missing loop segments would affect the simulation results.

While we agree that these loops may play a role in receptor flexibility and signaling in vivo, previous work (e.g., Wacker et al., 2013; McCorvy et al., 2018) suggests that the orthosteric site and G-biased signaling are primarily determined by residues within the transmembrane core. ICL3 is thought to selectively recognize G_q proteins.

Modelling such long loops is non-trivial and in this particular case incurs the theoretical risk that the loop might interact inappropriately with the protein in the absence of the complete intracellular environment. We instead chose to cap the intracellular end of TM5 and TM6 with acetylamide and methylamide groups at the N- and C-termini, respectively, as has been commonly done by other authors (e.g. Wallach 2023)

3. P3, line 67, the authors state that "(R)-69, which is biased to G protein pathway activation (Emax=87% relative to 5-HT) without any β-arrestin2 recruitment". This is incorrect. Although compound (R)-69 has been described as G protein-biased, it showed potent activation of the β-arrestin signaling.

We agree that the original phrasing was inaccurate and overstated the absence of β-arrestin2 recruitment. In the Chen et al. (2023) study, (R)-69 is indeed G protein-biased at the 5-HT2A receptor, but β-arrestin2 recruitment was also detectable at higher concentrations. In particular, their Extended data figure 8 shows that (R)-69 induces a concentration-dependent βArr2 response, albeit with lower potency compared to 5-HT. Therefore, we have revised the

sentence on P3, line 67 to more accurately reflect these findings. We thank the reviewer for prompting this clarification.

4. Figure 1, compounds IHCH-7086 and (R)-69 have designated chirality, such information should be presented.

This has now been addressed in the manuscript.

5. Figure 3: The color scheme lacks sufficient contrast. Consider using both color and shape coding for clearer distinction.

We revised all figures to improve contrast and readability, and added additional labels to facilitate easier distinction.

6. The names of amino acid residues are inconsistently represented, with a mix of three-letter and single-letter codes used.

We thank the reviewer for bringing this inconsistency to our attention. We now adapted the single-letter code followed by both the residue number and the Ballesteros-Weinstein numbering in the manuscript text. Conversely, the one-letter code followed by only the BW numbering was used in figures for the sake of clarity.
