# Supplementary material for: Molecular dynamics study of differential effects of serotonin-2A-receptor (5-HT2AR) modulators
Source: PLoS Comput Biol. 2025 Sep 3;21(9):e1013000. doi: 10.1371/journal.pcbi.1013000 (PMC12443254; doi:10.1371/journal.pcbi.1013000)
Supplement: Attachment — Submitted filename: response_to_reviewers.docx [file pcbi.1013000.s013.docx]

***Reviewers' comments:***

*Reviewer's Responses to Questions*

**Reviewer #2**: The authors have addressed my comments and concerns satisfactorily, and the manuscript can be recommended for publication. I appreciate the effort they put into implementing my critique and suggestions, and the thoroughness with which they answered my questions. Although there were some points of disagreement, which is entirely expected in the scientific process, the revisions reflect meaningful engagement with the review. The manuscript has significantly improved as a result, making a valuable contribution to our understanding of the 5-HT2A receptor.

*We sincerely thank the reviewer for the constructive feedback throughout the peer review process. We are grateful that the reviewer finds our responses satisfactory and that the manuscript is now recommended for publication.*

**Reviewer #3**: The authors have adequately addressed all my concerns in the revised manuscript, and I recommend its acceptance for publication.

However, prior to final publication, I suggest including a brief discussion of a very recent study (https://doi.org/10.1016/j.neuron.2025.06.012) in the Introduction section. This work challenges the notion of direct psychedelic-TrkB binding, demonstrating no observable TrkB activation across a comprehensive panel of 41 psychedelics. Given the significance of this finding to the broader field, a mention of this work—even as a point of ongoing debate—would be necessary.

*We would like to express our gratitude to the reviewer for the careful evaluation of our revised manuscript. We appreciate that the reviewer shared this recent study with us. We added a brief discussion of this work to our introduction.*
